# Skew-Circulant-Matrix-Based Harmonic-Canceling Synthesizer for BIST Applications

**DOI:** 10.3390/s22082884

**Published:** 2022-04-09

**Authors:** Guillermo G. Garayar-Leyva, Hatem Osman, Johan J. Estrada-López, Oscar Moreira-Tamayo

**Affiliations:** 1 Electrical and Computer Engineering Department, Texas A&M University, College Station, TX 77843, USA; omoreira@tamu.edu; 2 Silicon Labs, Austin, TX 78701, USA; hatem.osman@silabs.com; 3 Physics and Engineering, Westmont College, Santa Barbara, CA 93108, USA; jestradalopez@westmont.edu

**Keywords:** Built-In Self-Test, Harmonic-Canceling Filter, Skew-Circulant Matrix, CMOS, 180 nm

## Abstract

Testing is an important part of the design flow in the semiconductor industry. Unfortunately, it also consumes up to half of the production cost. On-silicon stimulus generators and response analyzers can be integrated with the Device-Under-Test (DUT) to reduce production costs with a minimum increment in power and area consumption. This practice is known as the Built-In Self-Test (BIST). This work presents a single-tone generator for BIST applications that is based on the Harmonic-Canceling (HC) technique. The main idea is to cancel or filter out the harmonics of a square-wave signal in order to obtain a highly pure sine wave. The design challenges of this technique are the precise implementation of irrational coefficients in silicon and the strong dependence of the output’s linearity on the coefficients’ precision. In order to reduce this dependence, this work introduces an irrational coefficient generator that is based on the recursive use of special matrices called skew-circulant matrices (SCMs). A complete study of the SCM-based HC synthesizer, its properties, and the proposed implementation in 180 nm CMOS technology are presented. The measured results show that the proposed HC synthesizer is able to filter out up to the 47th harmonic of a given square wave and to generate signals from 0.8 to 100 MHz with a maximum Spurious-Free Dynamic Range (SFDR) of 66 dB.

## 1. Introduction

**Figure 1:**
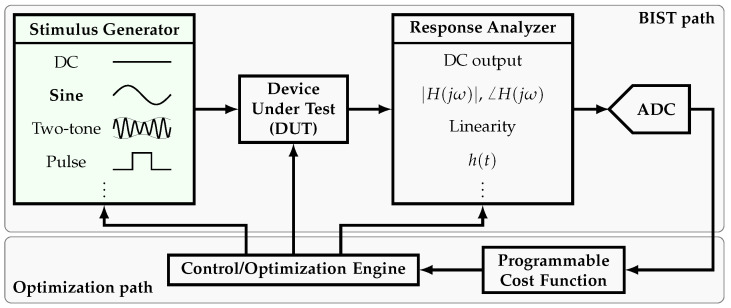
Simplified block diagram of a BIST and optimization systems.

The semiconductor industry has evolved significantly since its creation in the 1950s. Nowadays, testing has proven to be a decisive stage of the production flow. However, testing can consume as much as 55% of the production cost [1]. Consequently, adding on-chip, self-testing capabilities to the Device-Under-Test (DUT), provided by signal generators and response analyzers, has become a practical solution known as the Built-In Self-Test (BIST) approach. In order to make this an efficient solution, the required circuitry must be small in area and consume low power relative to the DUT. A block diagram of a BIST system and the complementary optimization system is shown in Figure 1. The BIST system consists of the stimulus generator, the response analyzer, and an Analog-to-Digital converter (ADC). In order to characterize the DUT, several stimuli can be made available, such as sine wave (single-tone) generators [2,3,4,5,6,7,8,9,10,11,12,13,14,15], two-tone generators [16,17], etc. Complementarily, in order to study the DUT response, several on-chip analyzers have been proposed such as spectrum analyzers [18,19,20,21], linearity analyzers [22,23,24], etc. Based on the BIST path output, the optimization path is able to take a decision and feed back the corresponding tuning signals into the BIST path.

This work focuses on the stimulus generator block, specifically, in the single-tone generator. In addition, it is an expanded version of a previous work [2]. For BIST applications, besides the low-area and power requirements, this block’s output must present a high linearity. For instance, in order to characterize a 10-bit ADC, a sinewave with Total Harmonic Distortion (THD) lower than −68 dB is required, which is challenging to obtain with a fully integrated system. Furthermore, technology scaling increases the design complexity due to the addition of nonlinearities and reduced voltage headroom.

**Figure 2:**
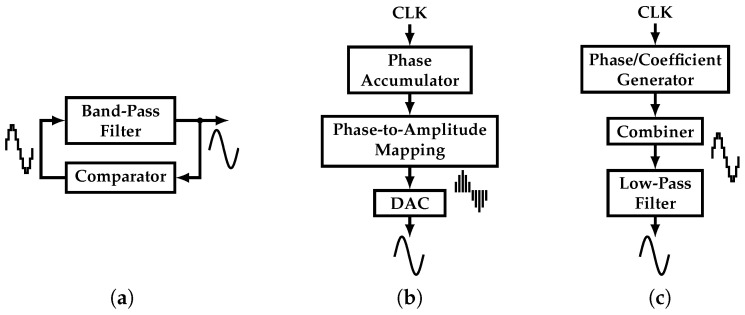
Different single-tone generators: (**a**) BPF-based oscillator, (**b**) DDFS, and (**c**) Harmonic-Canceling synthesizer.

Different approaches to tackle this challenge have been proposed. As presented in [3,4], a low-distortion, single-tone signal can be synthesized by a band-pass filter (BPF) in positive feedback with a multi-level comparator block, as shown in Figure 2a. Unfortunately, the output’s THD is directly proportional to the quality factor of the BPF and complexity of the comparator. This translates into a power hungry, large area filter. In addition, the BPF suffers from a limited frequency tuning range. In addition, the multi-level comparator is sensitive to process variations, introducing more distortion sources.

In order to expand the frequency tuning range, the Direct Digital Frequency Synthesizer (DDFS) is proposed in [5,6,7,8,9]. It can produce a highly linear tone based on a clock signal, and it usually is divided into a phase accumulator, a phase-to-amplitude mapping (P2AM) block, and a DAC (Figure 2b). Its digital nature makes it robust to technology scaling. However, this approach suffers from a high power consumption due to the P2AM block, which is typically based on a Read-Only Memory (ROM).

On the other hand, Harmonic-Canceling (HC) synthesizers appear as a solution with superior power consumption and tuning range capabilities compared to the previous approaches [10,11,12,13,14,15,16]. It uses phase-shifted versions of a clock signal which are scaled by coefficients that belong to a half-period sine function, i.e., irrational coefficients. It presents a phase generator, a coefficient generator (CG), and a combiner, as shown in Figure 2c. Previous works have integrated the required irrational coefficients in silicon by using ratios of integer numbers [11,12,13,14,15,16]. The main drawback of this approach is the trade-off between output linearity and process-variation sensitive coefficient precision. This imposes the use of calibration techniques that add to the system’s complexity.

This work proposes a programmable, high-order HC synthesizer that presents an irrational coefficient generator that ideally produces high-precision coefficients with no calibration scheme. This coefficient generator exploits the properties of a special family of matrices called skew-circulant matrices (SCMs) in a recursive approach. Its programmability allows the user to select the position of the non-cancelable harmonics, which are intrinsic to any HC synthesizer, in order to meet different linearity requirements. On the other hand, its high order reduces the complexity of the required additional low-pass filter (LPF) [12,13,15].

The document is organized as follows. Section 2 presents the mathematical background and classification of the HC synthesizer. Section 3 shows the relationship between the HC synthesizer and the SCMs. In addition, it presents the proposed SCM-based HCF and its properties. Next, a detailed circuit implementation is shown in Section 4. Section 5, Section 6 and Section 7 show the measurement results of the fabricated synthesizer, discussion, and conclusions, respectively.

## 2. Harmonic-Canceling Filter

**Figure 3:**
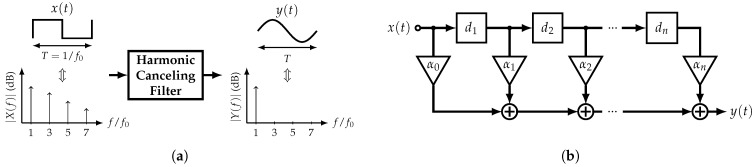
Harmonic-Canceling Filter: (**a**) Main concept and (**b**) a generic block diagram.

The main concept behind this type of filter is the rejection of the harmonics of a specific input signal in order to obtain a highly pure sine wave at its output; hence, they can be used as single-tone generators. Due to their frequency behavior, digital nature, and not very complex implementation, square waves (SWs) are considered as the filter’s input in this work. Figure 3a presents the operation of an ideal HCF when it is driven by a 50% duty cycle SW with fundamental angular frequency $\omega_{0}\;=\;2\pi f_{0}$. The ideal output corresponds to a pure single-tone signal with period $T\;=\;1/f_{0}$. Based on the Fourier series theory, any periodic signal f(t) can be expressed as

(1) $$f\left(t\right)=\frac{A_{0}}{2}+\sum_{k=0}^{\infty }\left[A_{k}\cos\left(k\omega_{0}t\right)+B_{k}\sin\left(k\omega_{0}t\right)\right]$$

where $A_{k}$ and $B_{k}$ are the Fourier coefficients, and $\omega_{0}$ is the fundamental angular frequency of f(t).

If *M* periodic signals f(t) with weight $\alpha_{i}$, delay $d_{i}\;=\;\theta_{i}/\omega_{0}$, and no DC component are considered, the Fourier series of the resultant signal $f_{eq}\left(t\right)$ is

(2) $$f_{eq}\left(t\right)=\sum_{i=0}^{M-1}\left[\alpha_{i}f\left(t+\frac{\theta_{i}}{\omega_{0}}\right)\right]=\sum_{i=0}^{M-1}\left[X_{k}\cos\left(k\omega_{0}t\right)+Y_{k}\sin\left(k\omega_{0}t\right)\right]$$

where its Fourier coefficients are

(3) $$X_{k}=\sum_{i=0}^{M-1}\alpha_{i}\left[A_{k}\cos\left(k\theta_{i}\right)+B_{k}\sin\left(k\theta_{i}\right)\right]$$

(4) $$Y_{k}=\sum_{i=0}^{M-1}\alpha_{i}\left[B_{k}\cos\left(k\theta_{i}\right)-A_{k}\sin\left(k\theta_{i}\right)\right]$$

The goal of an HCF is to eliminate $X_{k}$ and $Y_{k}$ for k≥2. In order to achieve this, from (3) and (4), there are two available degrees of freedom: $\alpha_{i}$ and $\theta_{i}$. Depending on which one is fixed, there are two approaches to implement an HCF, which are the constant-amplitude HCF and the constant-delay HCF. Figure 3b shows a generic block diagram of an HCF which resembles a Finite Impulse Response (FIR) filter.

### 2.1. Constant-Amplitude HCF

**Figure 4:**
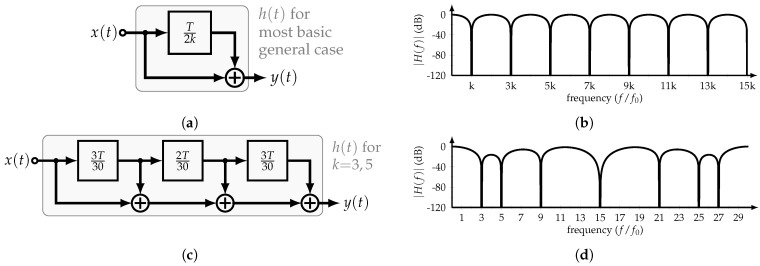
Constant–amplitude HCF: (**a**) Block diagram and (**b**) transfer function of most basic implementation, and (**c**) block diagram and (**d**) transfer function of HCF with rejection of 3rd and 5th harmonic and their odd multiples.

The basic implementation and transfer function |H(f)| of the constant-amplitude or time-mode HCF are shown in Figure 4a,b, respectively. Its transfer function is equal to

(5) $$\left|H(f)\right|=2\left|\cos\left(\pi f\tau_{D}\right)\right|$$

where *f* is the frequency in Hz. Interestingly, with only one delay element and a summer, the filter’s transfer function presents nulls at odd multiples of $1/2\tau_{D}$. Therefore, considering the input x(t) with period *T*, and setting $\tau_{D}\;=\;T/2k$, it is possible to cancel the odd multiples of the input’s *k*-th harmonic. Consequently, by adding several time delays in a specific manner, more harmonics can be canceled. For example, if the 3rd and 5th harmonics are to be suppressed, the corresponding HCF transfer function is

(6) $$\left|H(f)\right|=\left|\prod_{k=3,5}\cos\left(\pi f\frac{T}{2k}\right)\right|=\frac{1}{2}\left|\cos\left(\pi f\frac{2T}{30}\right)+\cos\left(\pi f\frac{8T}{30}\right)\right|$$

Figure 4c,d show the block diagram and transfer function of this HCF, respectively. As expected, the odd multiples of the 3rd and 5th harmonics are canceled.

Unfortunately, the number of harmonics to be canceled is inversely proportional to the size of the required delay unit. For instance, a delay unit of T/1890 is needed to suppress the odd multiples of the 3rd, 5th, and 9th harmonics. This trade-off turns the constant-amplitude HCFs into an impractical solution for high-speed applications. Nonetheless, some solutions have combined constant-amplitude HCFs with passive filters and optimization algorithms to tackle this problem [10].

### 2.2. Constant-Delay HCF

**Figure 5:**
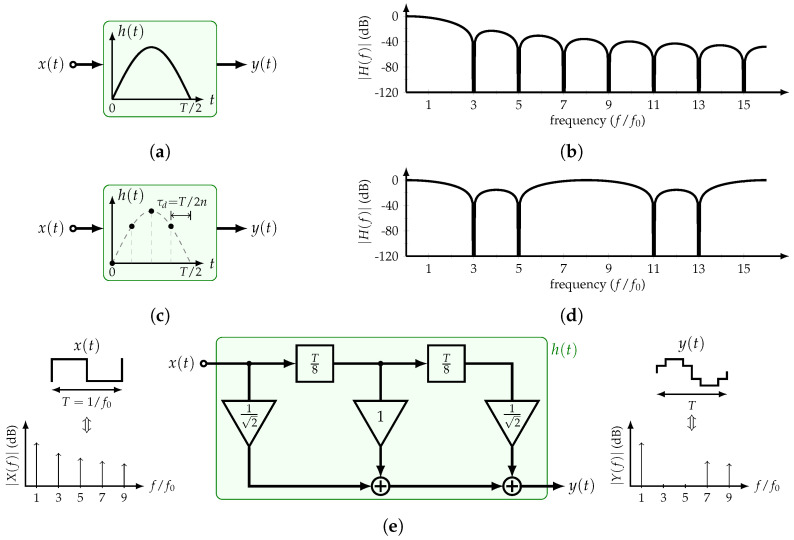
Constant–delay HCF: (**a**) Block diagram and (**b**) transfer function of the half-sine HCF; (**c**) block diagram, (**d**) transfer function, and (**e**) implementation of the 4-tap sampled half-sine HCF.

This type of filter is based on the concept of half-sine impulse response filters, which is shown in Figure 5a and was first proposed by [25]. Its transfer function is expressed as

(7) $$H\left(f\right)=\frac{2}{f_{0}}\frac{\cos\left(\frac{\pi }{2}\frac{f}{f_{0}}\right)}{1-{\left(\frac{f}{f_{0}}\right)}^{2}}$$

and is plotted in Figure 5b. This filter is able to suppress all the odd harmonics of the fundamental frequency $f_{0}\;=\;1/T$ of the SW input x(t) with period *T*, providing a highly pure tone as its output.

Recent publications have proposed practical implementations of this type of filters that used sampled versions of the half-sine impulse response [11,12,13,14,15,16]. If *n* samples of the impulse response are taken every $\tau_{d}\;=\;T/2n$, the filter is able to suppress all the input’s odd harmonics except those located at $\left(2ln\pm 1\right)f_{o}$ for l=1,2,…. Every sample corresponds to a tap coefficient $\alpha_{k}$ expressed as

(8) $$\alpha_{k}=h\left[k\right]=\sin\left(\frac{k\pi }{n}\right),\;k=0,1,\ldots ,n-1$$

This filter is also known as the n-tap HCF. Its transfer function is equal to

(9) $$\left|H(f)\right|=\frac{\cos\left(\frac{\pi }{2}\frac{f}{f_{0}}\right)\sin\left(\frac{\pi }{n}\right)}{\cos\left(\frac{\pi }{n}\frac{f}{f_{0}}\right)-\cos\left(\frac{\pi }{n}\right)}$$

Figure 5c,d illustrate the sampled impulse response and the transfer function of the 4-tap HCF. It is clear that the transfer function is periodic with a period of $2nf_{0}\;=\;8f_{0}$. Furthermore, Figure 5e shows its block diagram, SW input, and staircase sine-wave output. Since $\alpha_{0}\;=\;0$, only three coefficients and two delay units are required. Note that an irrational coefficient is used, and the 7th and 9th harmonics are non-cancelable due to the sampling operation. If the non-cancelable harmonics are required to be pushed to higher frequencies, it is necessary to increase the number of taps. At this point, a simple passive filter can attenuate them.

As discussed in this section, the sampled half-sine or constant-delay HCFs present advantages with respect to the constant-amplitude HCFs. For comparison purposes, an HCF that suppresses the 3rd and 5th harmonics is considered. On the one hand, a constant-delay 4-tap HCF requires a time step of T/8 and two unique coefficients. On the other hand, a constant-amplitude HCF requires a time step of T/30. It is clear that the former can achieve the same performance with a larger time delay. However, this comes with the challenge of implementing irrational coefficients. Considering BIST applications that use moderate to high frequency ranges in the order of MHz, this work focuses on the constant-delay HCFs. In the next section, a recursive approach to implement this filter is presented.

## 3. Proposed SCM-Based HCF

### 3.1. Matrix Representation of the HCF

From this point, a sampled half-sine HCF or constant-delay HCF is simply referred to as HCF. As presented in previous sections, an *n*-tap HCF requires *n* input SWs and *n* tap coefficients. Considering a 50% duty-cycle SW $\phi_{i}\left(t\right)$ with period *T*, then the *n*-tap HCF needs *n* versions of $\phi_{i}\left(t\right)$ with a delay of $\tau_{D}\;=\;T/2n$ with respect to each other. These are referred to as the input phases and can be expressed as

(10) $$\phi_{i,k}=\phi_{i}\left(t-\frac{kT}{2n}\right),\;k=0,1,\ldots ,n-1$$

Note that this set of SWs is periodic and odd symmetric. Hence, $\phi_{i,k+2n}=\phi_{i,k}$ and $\phi_{i,k+n}=-\phi_{i,k}$.

On the other hand, the tap coefficients $\alpha_{k}$ are given by (8). For an even *n*, it holds that $\alpha_{0}=0$, $\alpha_{n/2}=1$ and $\alpha_{k}=\alpha_{n-k}$. In other words, the HCF is a linear phase FIR filter; i.e., it provides a constant input-to-output group delay of $\tau_{D}\cdot \left(n/2\right)$. For this specific case, the HCF’s output $\phi_{o,n/2}$ can be defined as

(11) $$\phi_{o,\frac{n}{2}}=\sum_{k=0}^{n-1}\phi_{i,k}\alpha_{k}$$

Assuming that *n* outputs with a group delay ranging from 0 to (n−1) are required, the system can be expressed in matrix form as

(12) $$\left[\begin{array}{c} \phi_{o,0}\\ \vdots \\ \phi_{o,n/2-1}\\ \phi_{o,n/2}\\ \phi_{o,n/2+1}\\ \vdots \\ \phi_{o,n-1} \end{array}\right]=\left[\begin{array}{ccccccc} 1 & \alpha_{n/2-1} & \cdots & 0 & \cdots & -\alpha_{n/2-2} & -\alpha_{n/2-1}\\ \vdots & \vdots & \vdots & \vdots & \vdots & \vdots & \vdots \\ \alpha_{1} & \alpha_{2} & \cdots & \alpha_{n/2+1} & \cdots & \alpha_{1} & 0\\ 0 & \alpha_{1} & \cdots & 1 & \cdots & \alpha_{2} & \alpha_{1}\\ -\alpha_{1} & 0 & \cdots & \alpha_{n/2-1} & \cdots & \alpha_{3} & \alpha_{2}\\ \vdots & \vdots & \vdots & \vdots & \vdots & \vdots & \vdots \\ -\alpha_{n/2-1} & -\alpha_{n/2-2} & \cdots & 0 & \cdots & \alpha_{n/2-1} & 1 \end{array}\right]\times \left[\begin{array}{c} \phi_{i,0}\\ \vdots \\ \phi_{i,n/2-1}\\ \phi_{i,n/2}\\ \phi_{i,n/2+1}\\ \vdots \\ \phi_{i,n-1} \end{array}\right]$$

or in compact notation,

(13) $$\Phi_{o}=A_{i}\Phi_{i}$$

where $\Phi_{i}$, $\Phi_{o}$, and $A_{i}$ are the input phase vector, output phase vector, and the coefficients matrix, respectively. Interestingly, $A_{i}$ corresponds to a special matrix type called Skew-Circulant Matrix (SCM).

A n×n SCM $S_{n}$ is a matrix that presents a right cyclic shift between each consecutive row and the sub-diagonal elements change of sign [26]. Consequently, it is completely defined by the elements of its first row as $S_{n}\;=\;scirc\left(s_{o},s_{1},\ldots ,s_{n-1}\right)$. Another feature of the SCMs is that their eigenvectors $y_{m}$ only depend on their order *n* and can be expressed as

(14) $$y_{m}={\left[1,e^{\frac{j\pi \left(1+2m\right)}{n}},\ldots ,e^{\frac{j\pi \left(1+2m\right)\left(n-1\right)}{n}}\right]}^{T},\;m=0,1,\ldots ,n-1$$

where *j* is the unit imaginary number and *T* is the transpose operator. In addition, the eigenvalues $\lambda_{m}$ of $S_{n}$ are

(15) $$\lambda_{m}=\sum_{k=0}^{n-1}s_{k}e^{\frac{jk\pi \left(1+2m\right)}{n}},m=0,1,\ldots ,n-1$$

Considering the eigenvalues and eigenvectors of $S_{n}$, its eigen decomposition is expressed as $S_{n}\;=\;U\Lambda U^{*}$, where $U=[y_{0}|y_{1}|\ldots |y_{n-1}]$, $\Lambda =diag(\lambda_{0},\lambda_{1},\ldots ,\lambda_{n-1})$ and $U^{*}$ is the conjugate transpose of U. Based on these properties, all SCMs of the same order *n* share the same eigenvectors; hence, the same matrix U.

### 3.2. HCF with Multi-Stage Open-Loop SCM-Based Coefficient Generator

As shown in (13), an *n*-tap HCF can be represented by an SCM $A_{i}$ such that

(16) $$A_{i}=scirc\left(s_{0},s_{1},\ldots ,s_{n-1}\right)$$

where $s_{k}\;=\;\cos\left(k\pi /n\right)$ for k=0,1,…,n−1. For this case, it is proven in Appendix A that the eigenvalues of $A_{i}$ are equal to

(17) $$\lambda_{m}=\left\{\begin{array}{cc} n/2 & ,m=0,n-1\\ 0 & ,\mathrm{otherwise} \end{array}\right.$$

Consider the normalized, even-order *n* SCM $\left[A_{i}\right]$, and its eigen decomposition

(18) $$\left[A_{i}\right]=\frac{A_{i}}{∥A_{i}∥}=U\Lambda_{i}U^{*}$$

where $∥A_{i}∥$ is the Euclidean norm of $A_{i}$. Furthermore, from (17), it follows that matrix $\Lambda_{i}\;=\;diag\left(1,0,\ldots ,0,1\right)$.

For practical implementations, the main drawback of $\left[A_{i}\right]$ is that its elements potentially can be irrational numbers. In order to avoid this, matrix A is defined such that

(19) $$A=scirc(s_{0}^{\prime },s_{1}^{\prime },\ldots ,s_{n-1}^{\prime })$$

where $s_{k}^{\prime }\;=\;\mathrm{sgn}\left(s_{k}\right)$, and sgn(x) is the sign function. In this fashion, A is an integer-coefficient SCM. In Appendix B, it is proven that the eigenvalues of A are given by

(20) $$\lambda_{m}^{\prime }={\left(-1\right)}^{m}\cot\left(\frac{\pi \left(1+2m\right)}{2n}\right),m=0,1,\ldots ,n-1$$

Its normalized version [A] presents an eigen decomposition equal to

(21) $$\left[A\right]=\frac{A}{∥A∥}=U\Lambda U^{*}$$

Interestingly, using (20), $\Lambda \;=\;diag(1,\epsilon_{1},\ldots ,\epsilon_{n-2},1)$ where $\epsilon_{m}\;=\;\lambda_{m}^{\prime }/\max(|\lambda_{m}^{\prime }\left|\right)\;<$ 1. Based on this property, and recalling that all SCMs of the same order *n* share the same eigenvectors, if *M* replicas of [A] are cascaded, then

(22) $${\left[A\right]}^{M}={\left(\frac{A}{∥A∥}\right)}^{M}=U\Lambda^{M}U^{*}$$

where $\Lambda^{M}=diag\left(1,\epsilon_{1}^{M},\ldots ,\epsilon_{n-2}^{M},1\right)$. Then

(23) $$\lim_{M\to \infty }{\left[A\right]}^{M}=U\Lambda_{i}U^{*}=\left[A_{i}\right]$$

**Figure 6:**
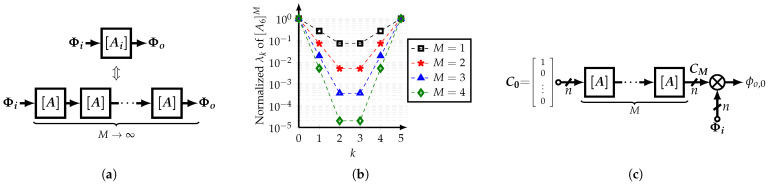
(**a**) Implementation of ideal HCF based on cascade of non-ideal SCMs, and (**b**) normalized eigenvalues of *M* SCMs $\left[A_{6}\right]$ in cascade, and (**c**) improved implementation.

Therefore, a cascade of *M* normalized, even-order *n*, integer-coefficient SCMs [A] can be used to approximate an irrational-coefficient SCM $\left[A_{i}\right]$, as shown in Figure 6a. In addition, Figure 6b shows the eigenvalues of the resultant SCM for different values of *M* and n=6. Note that the intermediate eigenvalues decrease as *M* increases. In other words, these intermediate eigenvalues can be considered as the error of the integer-coefficient SCM. It is important to note that the reason for using normalized matrices is that the outputs are bounded to the absolute magnitude of the input phases.

Since only one HCF’s output is required, the system architecture can be modified as shown in Figure 6c where the coefficients and phases generation processes are independent from each other. This improved approach allows that coefficients can be generated from a vector of DC signals $C_{0}\;=\;{\left[1,0,\ldots ,0\right]}^{T}$ and the phases present a faster path to the output, reducing potential phase errors. Nonetheless, this comes with the need for a combiner block.

Note that even if the challenge of using an irrational-coefficient-based SCM is met, it appears to be moved to the norm ∥A∥ since now, it can be an irrational number. It can be proven that ${∥A_{n}∥}^{-1}\;=\;\tan\left(\pi /2n\right)$. However, since this value affects the complete matrix A, it does not affect the coefficients’ relative ratio between each other; i.e., it can be considered as a gain error. In this work, the approximation ${∥A_{n}∥}^{-1}\;\approx \;8/5n$ is used.

### 3.3. HCF with Single-Stage Closed-Loop SCM-Based Coefficient Generator

**Figure 7:**
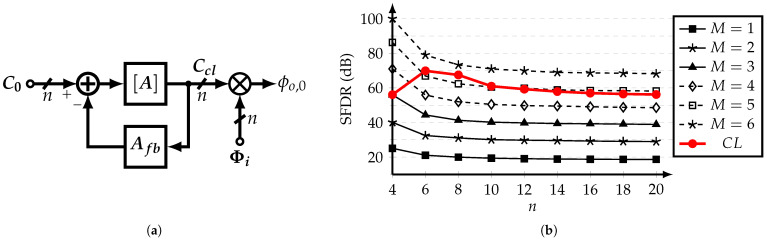
(**a**) Block diagram of the closed-loop CG, and (**b**) THD versus CG’s order *n*, for different *M*-stages open-loop CGs and single-stage closed-loop CG, with ${∥A∥}^{-1}\;=\;8/5n$.

From (23), it is implied that if M→∞, the outputs of $\left[A_{i}\right]$ and the cascade of ${\left[A\right]}^{M}$ are similar. This suggests the concept of the closed-loop SCM-based coefficient generator, which is presented in Figure 7a. Using the improved approach and at steady-state, the output vector $C_{cl}$ of the closed-loop coefficient generator is expressed as:

(24) $$C_{\mathrm{cl}}={\left(I+\left[A\right]A_{\mathrm{fb}}\right)}^{-1}\left[A\right]C_{0}$$

where $A_{\mathrm{fb}}\;=\;diag\left(0,1,1,\ldots ,1\right)$, and $C_{0}\;=\;{\left[1,0,\ldots ,0\right]}^{T}$. This is correct only if the ideal matrix norm ∥A∥ is used. The use of the approximation ${∥A_{n}∥}^{-1}\;\approx \;8/5n$ affects the coefficients’ relative ratio between each other; hence, it generates a systematic error.

In order to compare the performance of the multi-stage open-loop and single-stage closed-loop approaches, the spurious-free dynamic range (SFDR) of the filter’s output is evaluated using a system-level model. The SFDR is calculated as the ratio of the power of the fundamental frequency and the strongest cancelable harmonic up to the (2n−1)-th harmonic. Figure 7b shows the values of SFDR for different *n*-tap SCM-based HCFs using *M* open-stages and the closed-loop approach. It is observed that the closed-loop coefficient generator with ${∥A∥}^{-1}\;=\;8/5n$ is capable of achieving similar SFDR values as a 5-stage open-loop CG for n>6. Thus, the closed-loop CG with a non-ideal norm represents a less complex solution in comparison with the straightforward *M*-stage open-loop CG approach.

### 3.4. High-Order HCF

**Figure 8:**
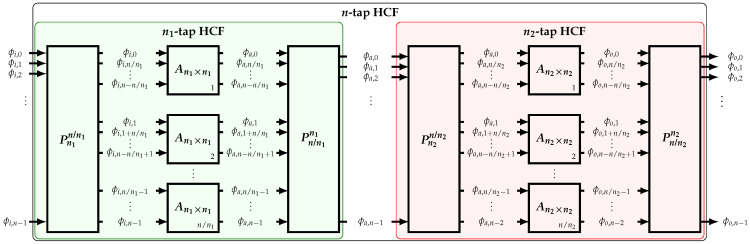
Implementation of a high-order HCF based on the cascade of two low-order HCFs.

As introduced in [16], a high-order *n*-tap HCF can be implemented by cascading lower-order $n_{1}$-tap and $n_{2}$-tap HCFs (Figure 8). A formal proof is shown in this section.

In order to use both HCFs, *n* input phases equally spaced by π/n are required such that $n\;=\;\mathrm{lcm}(n_{1},n_{2})$, where lcm(.) is the least common multiple operator. For the first stage to properly operate, $n/n_{1}$ parallel $n_{1}$-tap HCFs are needed. The phases are distributed based on a perfect shuffle permutation $P_{n_{1}}^{n/n_{1}}$ such that

(25) $$P_{r}^{s}=\left[\begin{array}{c} I_{n}\left(1:s:n,:\right)\\ I_{n}\left(2:s:n,:\right)\\ \vdots \\ I_{n}\left(s:s:n,:\right) \end{array}\right]$$

where n=s×r and $i_{n}$ is the n×n identity matrix. The MATLAB colon notation to designate submatrices is used. At the output of the $n_{1}$-tap HCFs, a perfect shuffle permutator $P_{n/n_{1}}^{n_{1}}$ is required to reorganize the output phases back to their original order. A similar process is done for the $n_{2}$-tap HCF. For each stage, these operations can be expressed as

(26) $$\begin{array}{c} \Phi_{a}=P_{n/n_{1}}^{n_{1}}\left(I_{n/n_{1}}⊗A_{n_{1}\times n_{1}}\right)P_{n_{1}}^{n/n_{1}}\Phi_{i}\\ \Phi_{o}=P_{n/n_{2}}^{n_{2}}\left(I_{n/n_{2}}⊗A_{n_{2}\times n_{2}}\right)P_{n_{2}}^{n/n_{2}}\Phi_{a} \end{array}$$

where ⊗ is the Kronecker product operator. For $X_{m\times n}\;=\;{\left(x_{ij}\right)}_{i=1,\ldots ,m;j=1,\ldots ,n}$ and $Y_{p\times q}\;=\;{\left(y_{hk}\right)}_{h=1,\ldots ,p;k=1,\ldots ,q}$, their Kronecker product is the mp×nq matrix given by

(27) $$X⊗Y=\left[\begin{array}{ccc} x_{11}Y & \cdots & x_{1n}Y\\ \vdots & \ddots & \vdots \\ x_{m1}Y & \cdots & x_{mn}Y \end{array}\right]$$

Based on the properties of the Kronecker product, (26) can be simplified to:

(28) $$\Phi_{o}=\left(A_{n_{2}\times n_{2}}⊗I_{n/n_{2}}\right)\left(A_{n_{1}\times n_{1}}⊗I_{n/n_{1}}\right)\Phi_{i}$$

As derived in Appendix C, matrix $\left(A_{n_{2}\times n_{2}}⊗I_{n/n_{2}}\right)\left(A_{n_{1}\times n_{1}}⊗I_{n/n_{1}}\right)$ is simply a scaled version of $A_{n\times n}$ if and only if $\gcd(n_{1},n_{2})\;>\;1$, and it is equal to

(29) $$A_{n\times n}=\frac{2}{\gcd\left(n_{1},n_{2}\right)}\times \left(A_{n_{2}\times n_{2}}⊗I_{n/n_{2}}\right)\left(A_{n_{1}\times n_{1}}⊗I_{n/n_{1}}\right)$$

where gcd(.) is the greatest common divisor operator. Hence, the cascade of the $n_{1}$-tap and $n_{2}$-tap HCFs is equivalent to an HCF of order $n\;=\;\mathrm{lcm}\left(n_{1},n_{2}\right)$ if and only if $n_{1}$ and $n_{2}$ have a common factor; i.e., $\gcd\left(n_{1},n_{2}\right)\;>\;1$.

### 3.5. Band-Pass HCF

As shown in Section 2, the objective of the half-sine HCF is to filter out all the harmonics of the input SW except its fundamental frequency. Nonetheless, it is possible to select the input’s *m*-th harmonic, which gives place to the band-pass HCF. Its impulse response $h_{m}\left(t\right)$ is given by

(30) $$h_{m}\left(t\right)=\sin\left(\frac{2\pi m}{T}t\right),\;0\leq t\leq \frac{T}{2}$$

**Figure 9:**
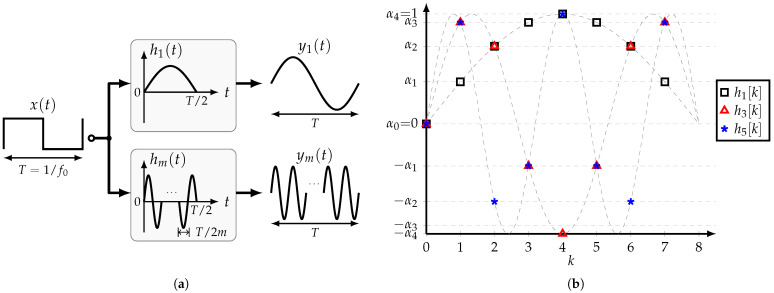
(**a**) Comparison between impulse responses of the basic and band-pass HCFs, and (**b**) impulse response of several band-pass HCFs for n=8.

Figure 9a shows a comparison between the basic and band-pass HCFs. If the *m*-th harmonic is to be bypassed to the output, then the HCF’s impulse response presents *m* half-sine segments.

For practical implementation, the impulse response is sampled at T/2n, where n>m to satisfy the Nyquist sampling theorem. Thus, for a given *n*-tap HCF, several band-pass HCFs can be obtained. Moreover, the sampled values $h_{m}\left[0,1,\ldots ,n/2\right]$ are all different if *m* and 2n are relatively prime, i.e., their greatest common divisor is 1. Figure 9b shows several band-pass HCFs for n=8. Note that $h_{m}\left[k\right]\;=\;\sin\left(mk\pi /n\right)$ is symmetric around k=n/2, and that the coefficients are similar for all the filters except that they present different orders and signs. Hence, assuming that the tap coefficients are available, it is possible to implement different band-pass HCFs by rearranging the tap coefficients accordingly.

## 4. Circuit Implementation

### 4.1. System Architecture

**Figure 10:**
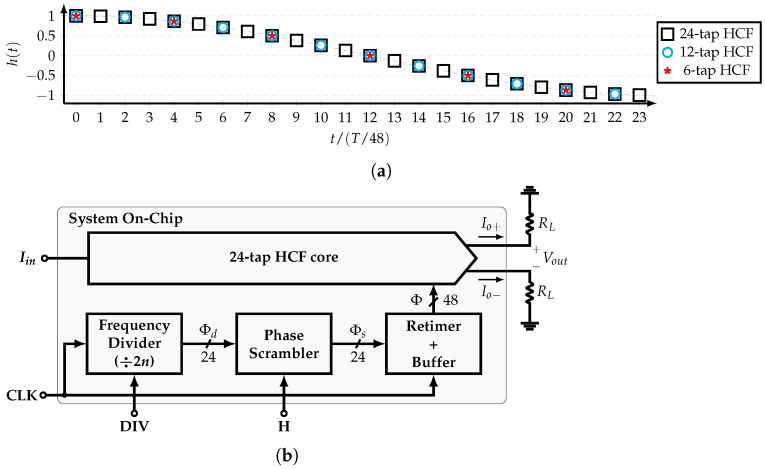
(**a**) Impulse response and (**b**) block diagram of system architecture.

In this work, a reconfigurable, SCM-based, 24-tap HCF is implemented. This filter is able to cancel up to the 47th harmonic of the SW signal ϕ(t) with frequency $f_{CLK}/48$. In other words, this HCF is used as a single-tone generator that produces a stepwise sine-wave differential current signal with frequency $f_{o}\;=\;max\left(f_{CLK}\right)/48$. Figure 10a shows its impulse response h(t), which corresponds to a cosine function cos(πk/24). It is noted that the coefficients related to $\phi_{2r-2}$, r=1,2,…,12 and $\phi_{4r-4}$, r=1,2,…,6 correspond to the 12-tap, and 6-tap HCFs, respectively. Thus, by selecting specific phases, the 24-tap, 12-tap, and 6-tap HCFs are available. This feature allows to extend the maximum frequency of the output signal to $f_{o}\;=\;max\left(f_{CLK}\right)/12$.

Figure 10b shows the block diagram of the complete system, which is divided in four main blocks: the frequency divider, the phase scrambler, the retimer and buffer, and the 24-tap HCF core. The frequency divider generates the 24 equally-spaced phases $\phi_{d}\left[0:23\right]$ from a clock signal CLK with programmable frequency division ratios in order to select between the 24-tap, 12-tap, and 6-tap HCFs. The phase scrambler allows for the rearrangement of the phases such that it can bypass the fundamental or the 5th input’s harmonic to its output. The 24-tap HCF core is divided in the CG and combiner. In order to achieve the required SCM order, 8-tap and 5-tap SCM-based CGs are used in cascade. All the required coefficients are generated using only one input DC current $I_{in}$. By means of a combiner, the system produces the differential output current $I_{o}$, which is converted to voltage by the load resistors $R_{L}$. Each block is presented in detail in the next subsections.

### 4.2. Frequency Divider

**Figure 11:**
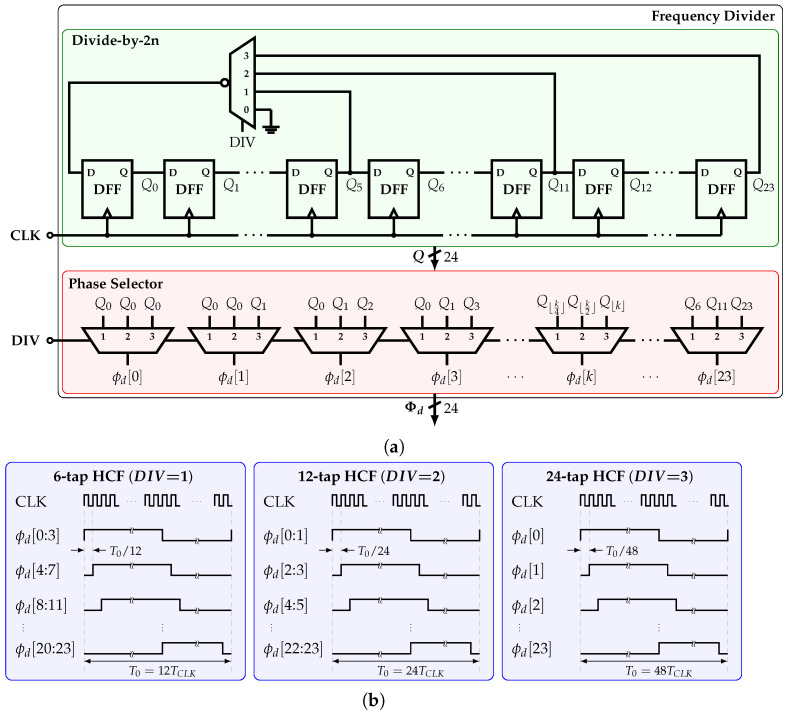
(**a**) Implementation and (**b**) output signals of the frequency divider.

The frequency divider (FD) is shown in Figure 11a. The 24 equally spaced phases are generated from the input clock signal CLK by a variable-length ring counter, which is based on a cascade of D flip-flops (DFFs). The outputs of this counter are $Q_{k}$, for k=0,1,…,23. Depending on the value of the input DIV∈{1,2,3}, the outputs $Q_{5}$, $Q_{11}$, or $Q_{23}$ are fed back to the input of the first DFF by an inverting feedback multiplexer, providing with a frequency division ratio of 12, 24, or 48, respectively.

The bus signal Q is connected to a phase selector with output $\Phi_{d}$. Depending on the value of DIV, each signal $\phi_{d}\left[k\right]$ is connected to $Q_{\left\lfloor k/4\right\rfloor }$, $Q_{\left\lfloor k/2\right\rfloor }$, or $Q_{\left\lfloor k\right\rfloor }$. Figure 11b shows the FD’s output phases pattern for each value of DIV. For example, for DIV=2, every two consecutive phases are connected; i.e., the corresponding coefficients are connected in parallel. In this fashion, the number of tap coefficients is kept constant for all available HCFs; hence, all the HCFs present the same output peak-to-peak amplitude.

### 4.3. Phase Scrambler

As shown in Section 3.5, the proposed HCF can be configured to bypass an input signal’s harmonic different from the fundamental frequency by rearranging its coefficients or phases. The latter approach is chosen due to its lower implementation complexity based on digital multiplexers.

**Figure 12:**
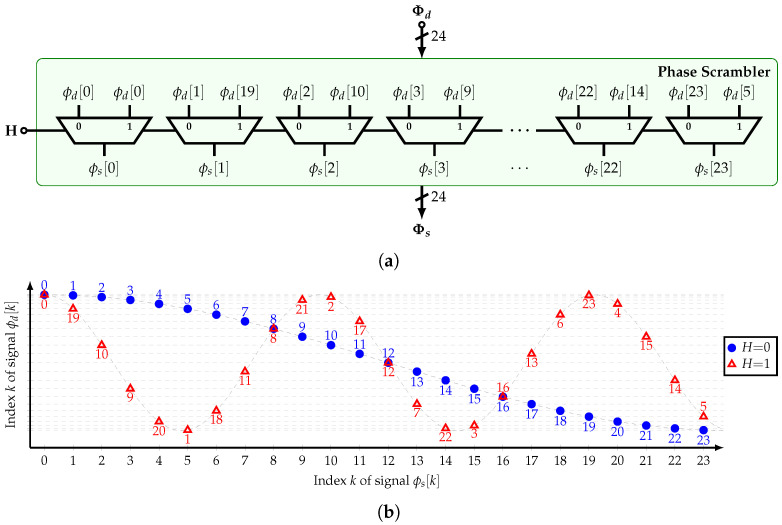
(**a**) Implementation and (**b**) input-to-output connections of the phase scrambler.

Figure 12a shows the implementation of the phase scrambler (PS). Depending on the value of H∈{0,1}, the fundamental frequency or the 5th harmonic of $\phi_{d}\left[k\right]$ are bypassed to the filter’s output, respectively. Note that 5 is coprime with 2n for the three available HCFs. Then, it is true that the tap coefficients of the bandpass HCF $h_{5}\left[k\right]\;=\;\cos\left(5k\pi /n\right)$ are similar to those of the low-pass HCF $h_{1}\left[k\right]\;=\;\cos\left(k\pi /n\right)$ but with a different order and sign. Figure 12b presents the input-to-output connections.

### 4.4. Retimer and Buffer

The required routing and operation of the phase selector and phase scrambler introduce phase errors. These are reduced by sampling the phase scrambler outputs $\phi_{s}\left[k\right]$ at the rising edge of the input clock CLK. This is done by an array of DFFs. Each of them provides an inverted version of each phase. The output of the retimer and buffer (R&B) is the bus Φ, where each signal ϕ[k]=−ϕ[k+24] for k=0,1,…,23. This work does not present any additional phase calibration scheme.

### 4.5. 24-Tap HCF Core

The required tap coefficients of the 24-tap HCF are generated by cascading the 8-tap and 6-tap CGs. Once these coefficients are available, they need to be combined with the phases accordingly in order to produce the system’s output. These operations are performed by the 24-tap HCF core.

The quarter-wave symmetry of the cosine function is used to reduce the implementation complexity of the CGs. In other words, by taking advantage of the SMC’s symmetry around $\alpha_{n/2}\;=\;1$, any given even-order n×n SCM $\left[A_{n}\right]$ can be expressed as an n/2×n/2 SCM $\left[A_{\mathrm{nr}}\right]$ such that

(31) $$\left[A_{\mathrm{nr}}\right]={∥A_{n}∥}^{-1}\left[\begin{array}{cccccc} 1 & 2 & 2 & \cdots & 2 & 2\\ 1 & 2 & 2 & \cdots & 2 & 1\\ \vdots & \vdots & \ddots & \ddots & \ddots & 0\\ 1 & 2 & 2 & 1 & \ddots & \vdots \\ 1 & 2 & 1 & 0 & \cdots & 0\\ 1 & 1 & 0 & 0 & \cdots & 0 \end{array}\right]$$

This reduced matrix contains the information related to only one quadrant of the cosine function. Using this property, matrix $\left[A_{8}\right]={∥A_{8}∥}^{-1}scirc\left(1,1,1,1,0,-1,-1,-1\right)$ can be reduced to

(32) $$\left[A_{8r}\right]=\frac{A_{8r}}{∥A_{8}∥}=\frac{1}{5}\left[\begin{array}{cccc} 1 & 2 & 2 & 2\\ 1 & 2 & 2 & 1\\ 1 & 2 & 1 & 0\\ 1 & 1 & 0 & 0 \end{array}\right]$$

In addition, $\left[A_{6}\right]\;=\;{∥A_{6}∥}^{-1}scirc\left(1,1,1,0,-1,-1\right)$ can be reduced even further, considering that it produces the coefficients 0.5 and 1(=0.5×2). Then

(33) $$\left[A_{6r}\right]=\frac{A_{6r}}{∥A_{6}∥}=\frac{4}{15}\left[\begin{array}{cc} 2 & 2\\ 1.5 & 2 \end{array}\right]$$

**Figure 13:**
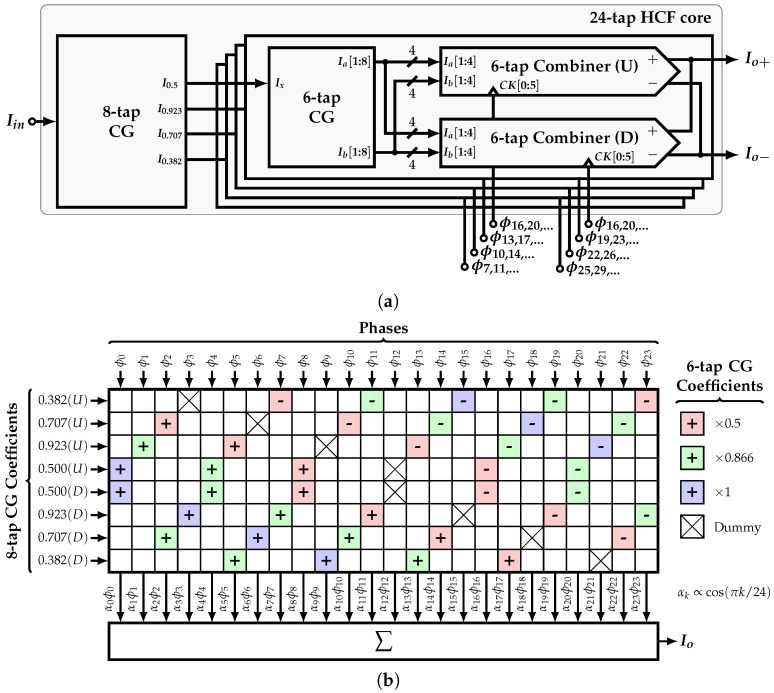
(**a**) Block diagram of the 24-tap HCF core and (**b**) phase-to-coefficient distribution.

Figure 13a shows the 24-tap HCF core block diagram. Based on the improved implementation presented in Section 3.3, input vector $C_{0}\;=\;\left(1,0,\ldots ,0\right)$ is used; i.e., a single input current $I_{in}$ is required to generate all the current-mode coefficients. The 8-tap CG implements the reduced SCM $\left[A_{8r}\right]$. It produces four output currents whose relative ratios with respect to each other correspond to the coefficients 0.5, 0.923, 0.707, and 0.382. Each of these outputs is connected to four 6-tap CGs, which in turn implement the SCM $\left[A_{r6}\right]$ and produce eight replicas of the currents $I_{a}$ and $I_{b}$ such that $I_{a}:I_{b}\;=\;1:0.866$.

The connection between the phases and coefficients is shown in Figure 13b. The absolute value and sign of the coefficients related to the 6-tap CG are color-coded. Each of them are scaled in the shown order by the 8-tap CG coefficients associated with each row. Moreover, each row shows the order of the phases connected to each 6-tap combiner unit. It is important to mention that the time delay between two consecutive combiner subcells of each row is 4T/48=T/12, that is, the unit delay of the 6-tap HCF, whereas the time delay between each row and the one below is 3T/48=T/16, which is the unit delay of the 8-tap HCF. In this way, all the phases present the same load, which reduces the systematic phase mismatch that limits the filter’s performance. Next, the resultant coefficient $\alpha_{k}$ corresponding to the sum of elements of the *k*-th column is multiplied by the corresponding phase. Finally, the output $I_{o}$ is equal to the sum of all $\alpha_{k}\phi_{k}$ products.

**Figure 14:**
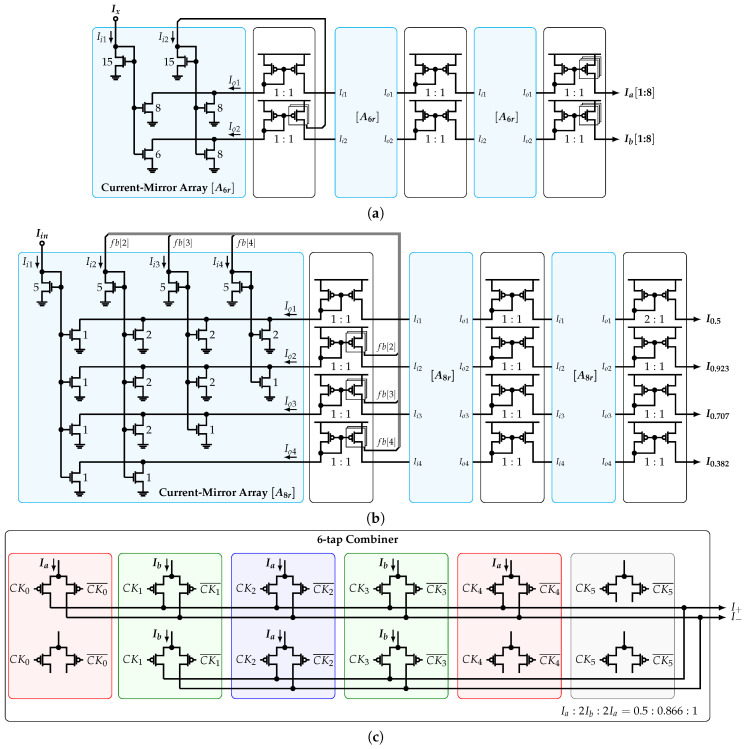
Circuit-level implementation of (**a**) 6-tap CG, (**b**) 8-tap CG, and (**c**) 6-tap combiner unit.

The circuit-level implementation of the 6-tap CG is shown in Figure 14a. It implements a cascade of three stages of matrix $\left[A_{6r}\right]$ along with its norm $∥A_{6}∥$ based on NMOS current mirrors (CMs). As presented in Section 3.3, the first stage is connected in a closed loop in order to achieve a filter’s output with SFDR>70 dB. In this work, the number of SCM stages is set to three due to a trade-off between the coefficient accuracy and area overhead. The PMOS CMs are used to transport the currents from stage to stage. The last PMOS CM provides eight copies of currents $I_{a}$ and $I_{b}$. The same approach is used to implement the 8-tap CG, as shown in Figure 14b. The implementation of the combiner unit is shown in Figure 14c. It is divided in twelve differential pairs and uses four copies of $I_{a}$ and $I_{b}$ that are connected as tail currents. In addition, six phases $CK_{0:5}$, each with its corresponding inverted version, are used to steer the input currents accordingly to the pattern presented in Figure 13b. If a negative sign is required, the differential clock is connected in opposite polarity. In this way, each section of the combiner inside the colored rectangles corresponds to each 6-tap coefficients; i.e., 0.5, 0.866, 1, 0.866, 0.5, and 0.

## 5. Measurement Results

**Figure 15:**
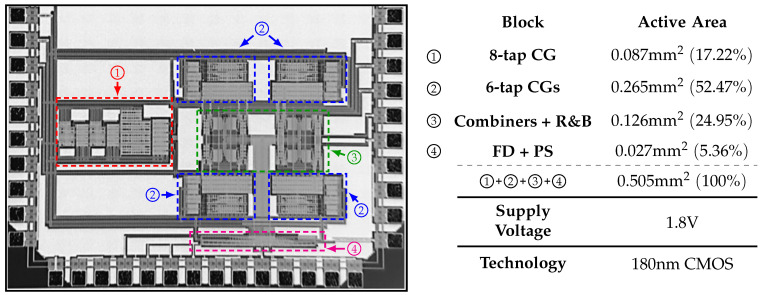
Micrograph of the fabricated single-tone generator.

The proposed single-tone generator is fabricated in 180 nm CMOS technology, operates with a supply voltage of 1.8 V, and occupies an area of 0.505mm${}^{2}$. Its micrograph is shown in Figure 15 along with the area occupied by each sector and its corresponding percentage with respect to the total area. The CGs occupy around 70% of the total area, since they are composed of a large amount of CMs. Furthermore, these CMs use large transistors in order to reduce their current–ratio mismatch, i.e., to improve the coefficients’ precision. In a CMOS process, the mismatch between two nominally identical transistors is inversely proportional to their channel length. Furthermore, recall that due to the recursive nature of the proposed solution, several identical blocks are required in order to obtain a specific SFDR, increasing the occupied area even further. In addition, the uncoupling of the phase generator from the coefficient generator contributes to the area cost.

**Figure 16:**
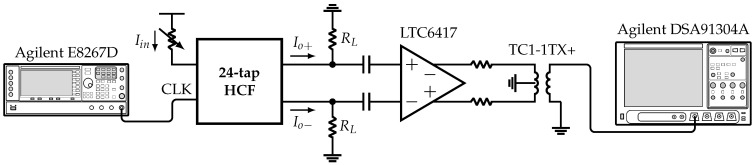
Measurement setup.

As presented in Section 4, the system incorporates six HCFs, which are selectable based on the value of the inputs n∈{6,12,24} and H∈{0,1}. The former selects between the 6-tap, 12-tap, or 24-tap HCFs, and the latter selects between the fundamental or 5th harmonic of the SW signal ϕ(t) with frequency $f_{CLK}/2n$. Figure 16 shows the measurement setup. The clock signal CLK with frequency $f_{CLK}$ is provided by an Agilent E8267D vector signal generator. The input current $I_{in}$ is set by a variable resistor. The differential output current $I_{o}$ is converted to voltage by the off-chip load resistors $R_{L}$. Next, this signal is buffered and converted to single-ended by the LTC6417 and TC1-1TX+, respectively. Finally, the resulting signal is analyzed using the Agilent DSA91304A Infiniium digital signal analyzer.

**Figure 17:**
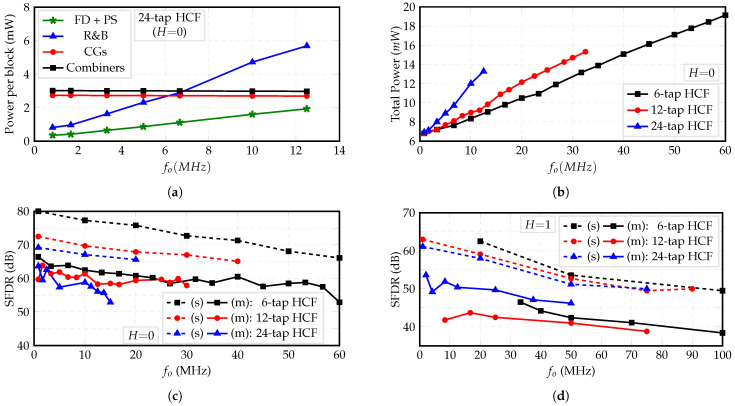
(**a**) Measured total power consumption of HCFs, (**b**) power consumption per block of 24-tap HCF for H=0, and simulated (s) and measured (m) SFDR for (**c**) H=0 and (**d**) H=1.

Figure 17a shows the measured power consumption of each block versus the output frequency $f_{o}$ of the 24-tap HCF when the fundamental frequency of ϕ(t), $f_{CLK}/48$, is of interest or H=0. Since the CGs only carry DC currents, its power consumption is independent of frequency. Furthermore, these currents are fed to the unit combiners, which steer them according to the pattern shown in Figure 13; hence, the combiner’s power consumption is also constant. Due to their digital nature, the FD, PS, and R&B blocks consume power proportional to the output frequency. In addition, Figure 17b shows the total power consumption of the 6-tap, 12-tap, and 24-tap HCFs versus the output frequency when H=0. These results show that the slopes of the curves are proportional to the filter’s order. This difference is mainly dictated by the fully digital blocks FD, PS, and R&B, especially the former, which enables only the required *n* DFFs.

The SFDR versus output frequency is shown in Figure 17c,d, for H=0 and H=1, respectively. It is noted that the SFDR decreases as the output frequency increases. This is due to the increasing phase error from the FD that causes even harmonics to show at the output [14]. Only the waveforms that present even harmonics with lower power than the odd cancelable harmonics are considered. Since the working frequency of the FD is greater for H=0 than for H=1, smaller SFDR values are obtained for H=1.

**Figure 18:**
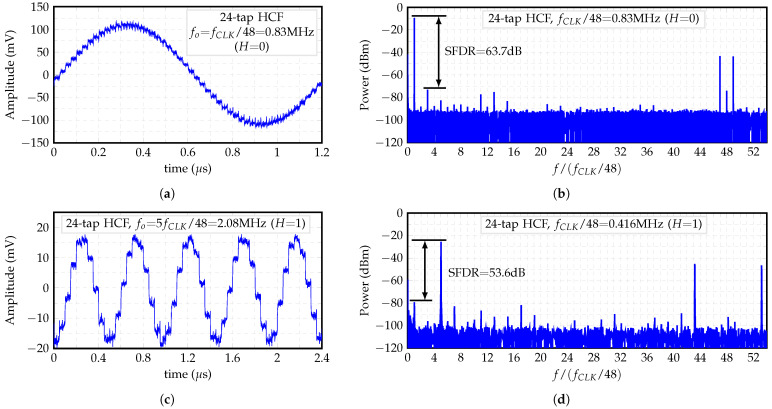
(**a**) Transient waveform and (**b**) PSD of HCF’s output for H=0, and (**c**) Transient waveform and (**d**) PSD of HCF’s output for H=1.

Figure 18a,b show the output’s waveform and power spectral density (PSD) of the 24-tap HCF, respectively, when H=0. The obtained staircase sine-wave waveform presents the first pair of non-cancelable harmonics at $47f_{o}$ and $49f_{o}$, which can be suppresed with a low-order passive LPF [12,13,15]. In addition, Figure 18c,d show the output’s waveform and PSD of the 24-tap HCF, respectively, when H=1. The first pair of non-cancelable harmonics is located at $43f_{o}$ and $52f_{o}$. Note that the carrier is located at $5f_{o}$.

Table 1 summarizes the performance of the six HCFs proposed in this work and compares them to previous works. The Figure of Merit (FoM) used in this work is given by

(34) $$\mathrm{FoM}=\frac{f_{o,max}\left(\mathrm{MHz}\right)\cdot 2^{\frac{{\mathrm{SFDR}}_{best}\left(\mathrm{dB}\right)}{6}}\cdot \mathrm{AF}\cdot \mathrm{FNCH}}{P_{total}\left(\mu W\right)\cdot A\left({\mathrm{mm}}^{2}\right)}$$

where $f_{o,max}$ is the maximum output frequency, ${\mathrm{SFDR}}_{best}$ is the highest measured SFDR, AF is the number of available filters, FNCH is the first non-cancelable harmonic, $P_{total}$ is the maximum total power consumption, and *A* is the area. This FoM is based on the one used by [13,14] with the addition that it accounts for the programmability and the harmonic-canceling range of the system. In this fashion, the number of implemented HCFs in the same area, i.e., the system’s area efficiency, is included in the FoM. On the other hand, recall that an external LPF is still required at the output of the HC-based generators due to the presence of the non-cancelable harmonics at $\left(2n\pm 1\right)f_{o}$. The order (and therefore, the complexity and power consumption) of the required external LPF is inversely proportional to the order *n* of the HCF. For this reason, it is relevant to include the FNCH in the FoM.

In summary, this work presents the only programmable HCF and the highest-order HCF. The 24-tap HCF allows the cancellation up to the 47th harmonic of the SW signal ϕ(t), which is the highest FNCH reported to the best knowledge of the authors. It also implements the first band-pass HCF. The proposed SCM-based HCFs provide SFDR and power consumption values comparable to previous works that use calibration techniques. For this work, the calculated FoM only includes the three HCFs when H=0. Considering the FoM values, this work performs better than most of the previous works except [13] only after it uses calibration.

## 6. Discussion

In the presented analysis, only ideal SCM elements and equally spaced SWs are considered. Therefore, it does not include non-idealities such as coefficients mismatch due to variations during fabrication or phase errors produced by the FD, PS, and R&B blocks. Under ideal conditions, as shown in Figure 7, the SFDR of the output signal increases as the number of SCM stages, *M*, increases, for a given HCF order *n*. Unfortunately, as presented in [16], non-idealities set a maximum limit for the output linearity. In other words, it is expected that the SFDR saturates and remains constant regardless of the number of SCM stages. This is reflected in the measured SFDR values, which are lower than expected from the ideal analysis. For this reason, an statistical analysis is required to optimize the HCF design in a future work. For instance, a model of the proposed HCF that considers the standard deviation of the CMs and the phase errors can be used to evaluate the trade-off between phase error, coefficient precision, and SFDR.

The use of a first-order approximation of the matrix norm ${∥A∥}^{-1}\;=\;8/5n$ is another source of SFDR limitation. Nonetheless, a better approximation requires the ratio of higher-integer numbers. For instance, consider the HCF of order n=6. Its ideal norm ${∥A_{i}∥}^{-1}\;=\;\tan\left(\pi /12\right)\;\approx \;0.2679$ is approximated as ${∥A∥}^{-1}\;=\;0.2666$. The next set of integer numbers, the ratio of which is closer to $∥A_{i}{∥}^{-1}$, is 15/56≈0.2678. The use of 15 and 56 in the matrix norm implementation implies the use of more unit transistors and a more complex device layout, i.e., more error sources that affect the SFDR.

In order to increase the output frequency range, the phase error produced by the FD, PS, and R&B blocks must be reduced. Note that these blocks operate at $2nf_{o}$. This is the main reason for the difference between the frequency ranges of the 6-tap, 12-tap, and 24-tap HCFs. In order to reduce the phase error in a future work, a delay error correction mechanism would be required. This can be provided by a Delay-Locked Loop (DLL) that generates the required phases with a negative feedback loop.

## 7. Conclusions

In this work, a harmonic-canceling single-tone synthesizer that uses an SCM-based coefficient generator for BIST applications is proposed. This coefficient generator produces irrational coefficients from integer numbers in a recursive approach with no calibration scheme. Measured SFDR values prove the effectiveness of the proposed SCM-based coefficient generator architecture, since they are comparable with those of previous works that use calibration. The selectable 24-tap, 12-tap, and 6-tap HCFs are implemented along with their band-pass versions. They cover a frequency range from 0.8 to 100 MHz and provide the highest number of operation modes and the highest first non-cancellable harmonic reported.

## Figures and Tables

**Table 1:** Performance comparison.

	Year	Tech.	$V_{\mathrm{DD}}$ (V)	Area (mm${}^{2}$)	Coefficient Generation	HCF Order	Bypassed Harmonic	$f_{o}$ (MHz)	SFDR/-THD * (dBc)	Power (mW)	FoM
									@ $f_{o}$ (MHz)	@ $f_{o}$ (MHz)	
This Work	2022	180 nm CMOS	1.8	0.505	SCM-based	6-tap	1st	0.8–60	66.4 @ 0.8	6.8 @ 0.8	1797
									52.9 @ 60	19.1 @ 60	
							5th	33–100	46.5 @ 33	6.1 @ 33	
									38.4 @ 100	8.7 @ 100	
						12-tap	1st	0.8–32	64 @ 0.8	6.8 @ 0.8	
									53 @ 32	15.3 @ 32	
							5th	8.3–75	43.7 @ 8.3	5.3 @ 8.3	
									38.8 @ 75	8.7 @ 75	
						24-tap	1st	0.8–12.5	63.7 @ 0.8	6.9 @ 0.8	
									54.6 @ 12.5	13.3 @ 12.5	
							5th	2–50	53.6 @ 2	5.1 @ 2	
									46.2 @ 50	10.2 @ 50	
[15]	2019	28 nm FDSOI	NR	0.011	VCCS + calib. + LPF	6-tap	1st	1–333	41.5 ${}^{†}$ @ 166.67	NR	-
									52 ${}^{‡}$ @ 166.67		
[16]	2017	130 nm CMOS	1.2–1.5	0.056	CM ratios	12-tap	1st	0.01–1	NR	4 (single-tone)	-
[14]	2017	130 nm CMOS	1.2–1.5	0.066	Unit-current switches + DEM	4-tap	1st	2	69 ${}^{‡}$	0.94	840
[13]	2015	180 nm CMOS	1.0–1.8	0.08	Resistor-ratios + calibration + LPF	6-tap	1st	150–850	50.5 ${}^{†}$ @ 150		
									60.3 ${}^{‡}$ @ 150	9.1 @ 150	698 ${}^{†}$
									47 ${}^{†}$ @ 750	57.2 @ 850	6642 ${}^{‡}$
									70 ${}^{‡}$ @ 750		
[12]	2015	180 nm CMOS	1.8	0.04	Capacitor ratios + LPF	8-tap	1st	1.11	77 *	3.24	938
[10]	2010	130 nm CMOS	1.2	0.186	N/A	N/A	1st	10	72 *	4	716

NR: Not reported, ^†^: without calibration or DEM, ^‡^: with calibration or DEM, *: -THD. VCCS: Voltage-controlled current source, DEM: Dynamic element matching.

## Data Availability

Not applicable.

## Abbreviations

The following abbreviations are used in this manuscript:

BIST	Built-In Self-Test
HC	Harmonic Canceling
HCF	Harmonic-Canceling Filter
SCM	Skew-Circulant Matrix
SFDR	Spurious-Free Dynamic Range
DUT	Device-Under-Test
ADC	Analog-to-Digital Converter
THD	Total Harmonic Distortion
BPF	Band-Pass Filter
DDFS	Direct Digital Frequency Synthesizer
P2AM	Phase-to-Amplitude Mapping
CG	Coefficent Generator
SW	Square-Wave
CMOS	Complementary Metal-Oxide Semiconductor
CM	Current-Mirror
FD	Frequency Divider
PS	Phase Scrambler
R&B	Retimer and Buffer
NR	Not Reported
VCCS	Voltage-Controlled Current Source
DEM	Dynamic Element Matching

## Appendix A. Eigenvalues of the Even-Order SCM with Ideal (Irrational) HCF Coefficients

Consider the even-order SCM $A_{i}\;=\;scirc\left(s_{0},s_{1},\ldots ,s_{n-1}\right)$, where $s_{k}\;=\;\cos\left(k\pi /n\right)$ for k=0,1,…,n−1. From (15), the eigenvalues $\lambda_{m}$ of $A_{i}$ are

(A1) $$\lambda_{m}=\sum_{k=0}^{n-1}\cos\left(\frac{k\pi }{n}\right)e^{\frac{jk\pi \left(2m+1\right)}{n}},m=0,1,\ldots ,n-1$$

Then

(A2) $$\lambda_{m}=\sum_{k=0}^{n-1}\left[\frac{e^{\frac{jk\pi }{n}}+e^{\frac{-jk\pi }{n}}}{2}\right]e^{\frac{jk\pi \left(2m+1\right)}{n}}=\frac{1}{2}\sum_{k=0}^{n-1}\left[e^{\frac{jk2\pi \left(m+1\right)}{n}}+e^{\frac{jk2\pi m}{n}}\right]$$

The two geometric series in (A2) can be expressed in closed form as

(A3) $$\sum_{k=0}^{n-1}e^{\frac{jk2\pi m}{n}}=\frac{1-e^{j2\pi m}}{1-e^{\frac{j2\pi m}{n}}}=\left\{\begin{array}{cc} n & ,m=0,\pm n,\pm 2n,\ldots \\ 0 & ,\mathrm{otherwise} \end{array}\right.$$

and

(A4) $$\sum_{k=0}^{n-1}e^{\frac{jk2\pi (m+1)}{n}}=\left\{\begin{array}{cc} n & ,m=-1,\pm (n-1),\pm 2(n-1),\ldots \\ 0 & ,\mathrm{otherwise} \end{array}\right.$$

Consequently, the eigenvalues of the even-order SCM $A_{i}$ are given by

(A5) $$\lambda_{m}=\left\{\begin{array}{cc} n/2 & ,m=0,n-1\\ 0 & ,\mathrm{otherwise} \end{array}\right.$$

## Appendix B. Eigenvalues of the Even-Order SCM with Non-Ideal (Integer) HCF Coefficients

Consider the even-order SCM $A\;=\;scirc(s_{0}^{\prime },s_{1}^{\prime },\ldots ,s_{n-1}^{\prime })$, where $s_{k}^{\prime }\;=\;\mathrm{sgn}\left(\cos(k\pi /n)\right)$ for k=0,1,…,n−1, and $\mathrm{sgn}\left(\cdot \right)$ is the sign function. From (15), the eigenvalues $\lambda_{m}^{\prime }$ of A are

(A6) $$\lambda_{m}^{\prime }=\sum_{k=0}^{n-1}\mathrm{sgn}\left(\cos\left(\frac{k\pi }{n}\right)\right)e^{\frac{jk\pi \left(2m+1\right)}{n}},m=0,1,\ldots ,n-1$$

Then

(A7) $$\lambda_{m}^{\prime }=\sum_{k=0}^{\frac{n}{2}-1}e^{\frac{j\pi k\left(2m+1\right)}{n}}-\sum_{k=\frac{n}{2}+1}^{n-1}e^{\frac{j\pi k\left(2m+1\right)}{n}}=\sum_{k=0}^{\frac{n}{2}-1}e^{\frac{j\pi k\left(2m+1\right)}{n}}-\left[\sum_{k=0}^{n-1}e^{\frac{j\pi k\left(2m+1\right)}{n}}-\sum_{k=0}^{\frac{n}{2}}e^{\frac{j\pi k\left(2m+1\right)}{n}}\right]$$

From (A7), the geometric series can be further reduced using their closed form. It follows that

(A8) $$\lambda_{m}^{\prime }=\frac{1-e^{\frac{j\pi \left(2m+1\right)}{n}\left(\frac{n}{2}\right)}}{1-e^{\frac{j\pi \left(2m+1\right)}{n}}}-\left[\frac{1-e^{\frac{j\pi \left(2m+1\right)}{n}\left(n\right)}}{1-e^{\frac{j\pi \left(2m+1\right)}{n}}}-\frac{1-e^{\frac{j\pi \left(2m+1\right)}{n}\left(\frac{n}{2}+1\right)}}{1-e^{\frac{j\pi \left(2m+1\right)}{n}}}\right]$$

Simplifying

(A9) $$\lambda_{m}^{\prime }=e^{\frac{j\pi \left(2m+1\right)}{2}}\left[\frac{e^{\frac{j\pi \left(2m+1\right)}{n}}+1}{e^{\frac{j\pi \left(2m+1\right)}{n}}-1}\right]$$

Therefore, the eigenvalues of the even-order SCM A are

(A10) $$\lambda_{m}^{\prime }={\left(-1\right)}^{m}\cot\left(\frac{\pi \left(2m+1\right)}{n}\right),m=0,1,\ldots ,n-1$$

## Appendix C. Equivalence between a Cascade of Lower Order HCFs and a Higher Order HCF

Consider Equation (28), which describes the cascade of two SCM-based HCFs of order $n_{1}$ and $n_{2}$. Since matrix $A_{n_{1}\times n_{1}}$ is an SCM, it is true that $A_{n_{1}\times n_{1}}⊗I_{n/n_{1}}$ is also an SCM with its first row elements upsampled by $n/n_{1}$. The same holds for $A_{n_{2}}\times n_{2}⊗I_{n/n_{2}}$. Their eigenvalues are given by (A1) and are equal to

(A11) $$\begin{array}{c} \lambda \left[A_{n_{1}\times n_{1}}⊗I_{n/n_{1}}\right]=\left\{\lambda_{0},\lambda_{1},\ldots ,\lambda_{n-1}\right\}\\ \lambda \left[A_{n_{2}\times n_{2}}⊗I_{n/n_{2}}\right]=\left\{\mu_{0},\mu_{1},\ldots ,\mu_{n-1}\right\} \end{array},$$

where

(A12) $$\begin{array}{c} \lambda_{m}=\left\{\begin{array}{cc} n_{1}/2 & ,\mathrm{if}\;m\in S_{11}\\ n_{1}/2 & ,\mathrm{if}\;m\in S_{12}\\ 0 & ,\mathrm{otherwise} \end{array}\right. \end{array}$$

(A13) $$\begin{array}{c} \mu_{m}=\left\{\begin{array}{cc} n_{2}/2 & ,\mathrm{if}\;m\in S_{21}\\ n_{2}/2 & ,\mathrm{if}\;m\in S_{22}\\ 0 & ,\mathrm{otherwise} \end{array}\right. \end{array}$$

where

(A14) $$S_{11}=\left\{kn_{1}:k\in Z,0\leq k\leq \frac{n}{n_{1}}-1\right\}$$

(A15) $$S_{12}=\left\{kn_{1}+\left(n_{1}-1\right):k\in Z,0\leq k\leq \frac{n}{n_{1}}-1\right\}$$

(A16) $$S_{21}=\left\{kn_{2}:k\in Z,0\leq k\leq \frac{n}{n_{2}}-1\right\}$$

(A17) $$S_{22}=\left\{kn_{2}+\left(n_{2}-1\right):k\in Z,0\leq k\leq \frac{n}{n_{2}}-1\right\}$$

Since matrices $\left(A_{n_{1}\times n_{1}}⊗I_{n/n_{1}}\right)$ and $\left(A_{n_{2}\times n_{2}}⊗I_{n/n_{2}}\right)$ are SCMs, their product is also an SCM. Furthermore, since all of them present the same size n×n, they all share the same eigenvectors. The eigenvalues $\lambda_{m}\mu_{m}$, m=0,1,…,n−1 of the resultant matrix are expressed as

(A18) $$\lambda_{m}\mu_{m}=\left\{\begin{array}{cc} n_{1}n_{2}/4 & ,\mathrm{if}\;m\in S_{11}⋂S_{21}\\ n_{1}n_{2}/4 & ,\mathrm{if}\;m\in S_{12}⋂S_{22}\\ n_{1}n_{2}/4 & ,\mathrm{if}\;m\in S_{11}⋂S_{22}\\ n_{1}n_{2}/4 & ,\mathrm{if}\;m\in S_{12}⋂S_{21}\\ 0 & ,\mathrm{otherwise} \end{array}\right.,$$

where $S_{11}⋂S_{21}\;=\;\left\{0\right\}$, $S_{12}⋂S_{22}\;=\;\left\{n-1\right\}$, and $S_{11}⋂S_{22}\;=\;S_{12}⋂S_{21}\;=\;⌀$ if and only if $\gcd(n_{1},n_{2})>1$. It follows that

(A19) $$\lambda \left[\left(A_{n_{2}\times n_{2}}⊗I_{n/n_{2}}\right)\left(A_{n_{1}\times n_{1}}⊗I_{n/n_{1}}\right)\right]=\left\{\begin{array}{cc} n_{1}n_{2}/4 & ,\mathrm{if}\;m=0,n-1\\ 0 & ,\mathrm{otherwise} \end{array}\right.$$

Equation (A19) implies that the resultant SCM matrix is a scaled version of matrix $A_{n\times n}$ such that

(A20) $$\frac{n_{1}n_{2}}{4}A_{n\times n}=\frac{n}{2}\left(A_{n_{2}\times n_{2}}⊗I_{n/n_{2}}\right)\left(A_{n_{1}\times n_{1}}⊗I_{n/n_{1}}\right)$$

(A21) $$A_{n\times n}=\frac{2n}{n_{1}n_{2}}\left(A_{n_{2}\times n_{2}}⊗I_{n/n_{2}}\right)\left(A_{n_{1}\times n_{1}}⊗I_{n/n_{1}}\right)$$

Since *n* phases equally spaced by π/n are required, such that $n\;=\;\mathrm{lcm}\left(n_{1},n_{2}\right)$, and considering that $\mathrm{lcm}\left(n_{1},n_{2}\right)\;=\;n_{1}n_{2}/\gcd\left(n_{1},n_{2}\right)$, $A_{n\times n}$ can be expressed as

(A22) $$A_{n\times n}=\frac{2}{\gcd(n_{1},n_{2})}\left(A_{n_{2}\times n_{2}}⊗I_{n/n_{2}}\right)\left(A_{n_{1}\times n_{1}}⊗I_{n/n_{1}}\right)$$
